# A Meta-Analysis on the Rate of Hepatocellular Carcinoma Recurrence after Liver Transplant and Associations to Etiology, Alpha-Fetoprotein, Income and Ethnicity

**DOI:** 10.3390/jcm10020238

**Published:** 2021-01-11

**Authors:** Darren J. H. Tan, Chloe Wong, Cheng Han Ng, Chen Wei Poh, Sneha Rajiv Jain, Daniel Q. Huang, Mark D. Muthiah

**Affiliations:** 1 Yong Loo Lin School of Medicine, National University of Singapore, Singapore 117597, Singapore; e0433903@u.nus.edu (D.J.H.T.); e0421800@u.nus.edu (C.W.); e0360747@u.nus.edu (C.H.N.); e0421865@u.nus.edu (C.W.P.); snehajain@u.nus.edu (S.R.J.); daniel_huang@nuhs.edu.sg (D.Q.H.); 2 Division of Gastroenterology and Hepatology, Department of Medicine, National University Hospital, Singapore 119074, Singapore; 3 National University Centre for Organ Transplantation, National University Hospital, Singapore 119074, Singapore

**Keywords:** deceased donor liver transplant, ethnicity, epidemiology, HCC recurrence, living donor liver transplant, meta-analysis

## Abstract

Hepatocellular carcinoma (HCC) recurrence after liver transplant is associated with a poor prognosis and significantly increases morbidity and mortality among liver transplant patients. Therefore, this meta-analysis aims to evaluate the overall prevalence of HCC recurrence following liver transplant. Medline and Embase databases were searched, and a meta-analysis of proportions was conducted. Observational studies reporting the prevalence of recurrent hepatocellular carcinoma (HCC) after liver transplant were included, with the analysis being stratified by adherence to Milan criteria, ethnicity, socio-economic status, alpha fetoprotein (AFP) levels, living donor vs. deceased donor, and the underlying aetiology of the liver disease. A meta-regression on the date of the study completion was also performed. Of a total 40,495 patients, 3888 developed an HCC recurrence. The overall prevalence of recurrent HCC was 13% (CI: 0.12–0.15). Patients beyond the Milan criteria (MC) were more likely to recur than patients within MC. Asian populations had the greatest prevalence of HCC recurrence (19%; CI: 0.15–0.24) when compared to Western (12%; CI: 0.11–0.13) and Latin American populations (11%; CI: 0.09–0.14). The prevalence of recurrent HCC was the highest in patients infected with hepatitis B virus (HBV) (18%; CI: 0.11–0.27) compared to other aetiologies. A higher AFP also resulted in an increased recurrence. This highlights interesting differences based on ethnicity, income, and aetiology, and further studies are needed to determine the reasons for the disparity.

## 1. Introduction

Liver transplantation (LT) is regarded as being the gold standard of treatment for patients with hepatocellular carcinoma (HCC), due to the curative ability of the treatment in eliminating both the tumour and the underlying liver disease [1,2,3]. Liver transplantation enables the restoration of liver function and is associated with improved survival rates when strict pretransplantation criteria are met [4], with recent evidence even suggesting a comparable survival to other therapies after the downstaging of tumours beyond criteria [5]. Despite the proven curative efficacy of LT, current estimates suggest a 15–20% recurrence rate for HCC after transplant [6,7,8,9].

The risk of recurrence of HCC in post-transplant patients is increased in recipients with a tumor burden exceeding the criteria for transplantation [10], aggressive tumour biology, suboptimal locoregional therapy prior to LT [11], and with the use of immunosuppressants in patients [12]. In particular, immunosuppression therapy, which is essential after transplantation to minimise organ rejection [13], can increase the risk of recurrent HCC [14]. Given the poor prognosis in recurrent HCC, with an estimated median survival of seven to 16 months [12], it is crucial to have a better understanding of post-LT HCC recurrence across a wide array of patients. The paucity of published data regarding recurrent HCC prevalence among ethnic groups, the underlying aetiology, and the economic levels of countries warrants further investigations since these factors may affect access to care and predispose one to recurrence. Hence, this meta-analysis aims to examine the prevalence of HCC recurrence overall and by subgroups such as ethnicity, incomes, and aetiology, so that differences can be identified and can potentially drive further investigation.

## 2. Methods

### 2.1. Search Strategy

This meta-analysis was performed using the Preferred Reporting Items for Systematic Reviews and Meta-Analyses (PRISMA) guidelines [15]. Embase and Medline databases were systematically searched for relevant articles from inception until 24 July 2020 using terms and keywords synonymous with “hepatocellular carcinoma”, “recurrence”, and “liver transplantation”. The full search strategy is attached in Table S1. Citations were then downloaded and reviewed in Endnote Reference Manager X9 (Clarivate Analytics, Philadelphia, PA, USA).

### 2.2. Study Selection and Eligibility Criteria

Studies were considered for inclusion if they met the following inclusion criteria: (1) patients who had HCC as the main indication for LT, and (2) patients who had available long-term follow-up data. Articles deemed potentially relevant were first screened by title and abstract, followed by the full text for inclusion by two pairs of independent authors (D.J.H.T. and C.W.; C.H.P. and S.R.J.). Only original articles and abstracts written in or translated into the English language were included. A quantitative methodology, such as retrospective and prospective cohort studies, as well as observational studies were included. Case reports, reviews, and commentary were excluded. The final inclusion of articles was based on consensus between the two authors.

### 2.3. Data Extraction and Outcomes

For each article, two authors (D.J.H.T. and C.W.) independently extracted data into a structured proforma. Population demographics including age, ethnicity, income level, transplant type, and adherence to Milan criteria, pre-transplant alpha-fetoprotein (AFP), and the proportion of patients that had undergone locoregional therapy (LRT) prior to transplant were collected for each study. Income levels were defined according to the definitions set by the World Bank [16]. The main outcome that was analysed was the HCC recurrence after liver transplant, which was defined with radiological findings alone or in combination with alpha-fetoprotein levels, depending on the included studies (Table S2). In line with current guidelines and studies on liver transplantation for HCC [9,17,18,19] all post-transplant instances of HCC were classified as recurrences regardless of the timeframe.

Ethnicity was analyzed by the predominant ethnicity in the country of study [20]. A subgroup analysis was considered for HCC recurrence across different ethnic groups derived from the countries of origin, in countries of varying income levels, and across different transplant types. When mean and standard deviation data were not reported, conversions of data were performed using existing methods [21,22]. 

### 2.4. Statistical Analysis and Quality Assessment

Data was extracted and analyzed using the *metaprop* and *metareg* functions on STATA (16.1 StataCorp LLC, Texas, TX, USA) [23,24,25]. The meta-analysis of proportions was conducted using a Freeman–Turkey double arcsine transformation for the stabilization of variance, and the Dersimonian and Laird random effects model [26] was used for the pooled analysis. Meta-regression was done with the restricted maximum likelihood model with the Knapp–Hartung variance estimator [24]. The risk of bias was independently assessed in the included cohort studies by two authors using the metric described by Hoy et al. [27].

## 3. Results

**Figure 1:**
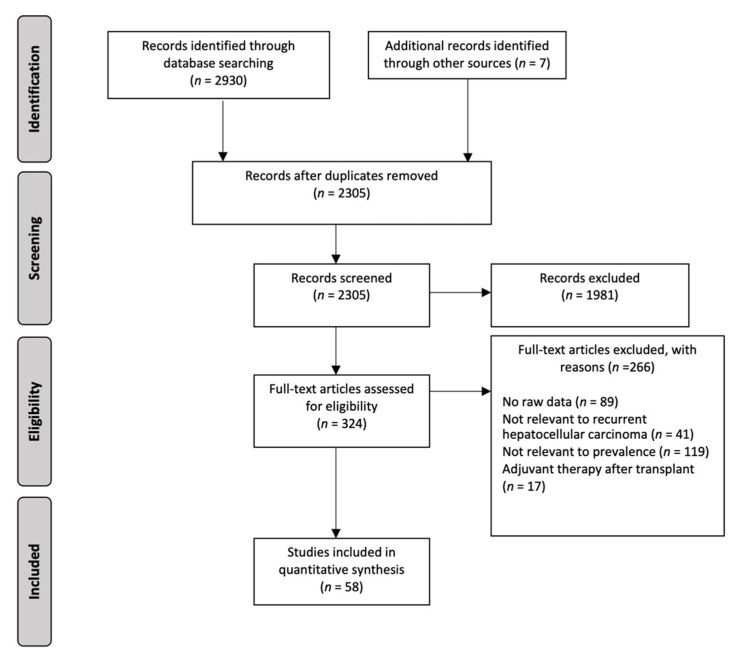
PRISMA Flow Diagram.

A total of 2930 records were identified through the combined search results, with 625 duplicates removed. 1981 manuscripts were excluded based on the title and abstract alone, and 324 were reviewed in a full text review. 58 articles ultimately met the inclusion criteria (Figure 1). From these included studies, there were 55 retrospective cohort studies and three prospective cohort studies. 40,495 patients were diagnosed with HCC and underwent liver transplant, of which there were 3888 recorded cases of HCC recurrence. A summary of the key characteristics, quality assessment, and reference list of the included studies is presented in Table S2.

### 3.1. Overall Prevalence of HCC Recurrence

**Figure 2:**
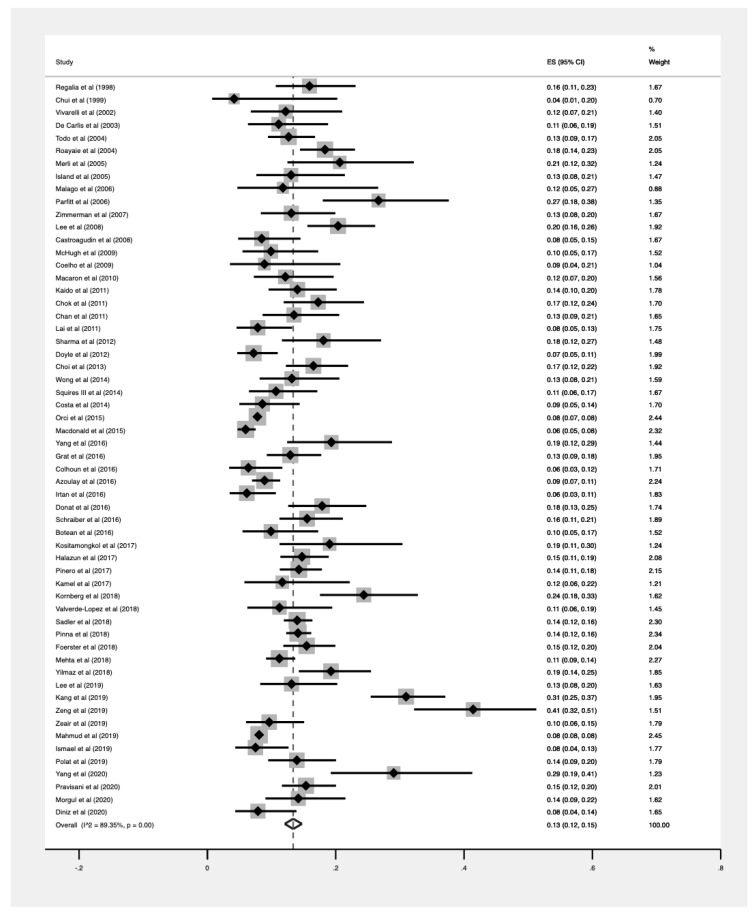
Pooled prevalence of all included studies.

**Figure 3:**
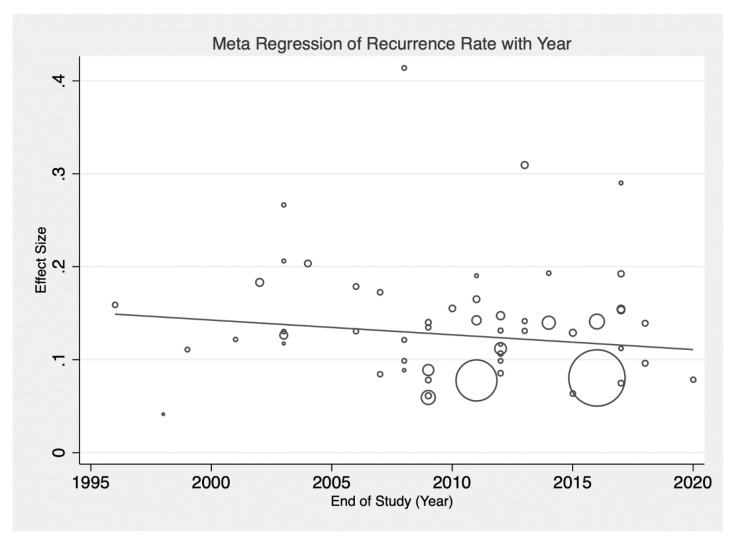
Meta-regression of HCC recurrence and study end date.

A summary of the pooled prevalence of HCC recurrence in different subgroups is presented in Table 1. A total population of 40,495 patients were diagnosed with HCC and underwent liver transplant. HCC recurrence was recorded in 3888 cases, and a pooled analysis revealed the overall prevalence to be 13% (CI: 0.12–0.15, Figure 2). The meta-regression analysis between the HCC recurrence and year of study completion revealed a decreasing trend of HCC recurrence, although without statistical significance (β = −0.015, SE = 0.002, *p* = 0.434, Figure 3).

### 3.2. Analysis by Pretransplant LRT

A meta-regression analysis between HCC recurrence and the proportion of patients that had undergone LRT prior to transplant was also conducted. Transarterial chemoembolisation did not have a significant effect on the prevalence of recurrent HCC (β = 0.053, SE = 0.079, *p* = 0.511). Radiofrequency ablation also did not affect HCC recurrence significantly (β = 0.033, SE = 0.120, *p* = 0.787).

### 3.3. Analysis by Pretransplant AFP

The prevalence of recurrent HCC was also stratified according to the mean AFP before transplant. In studies where the mean AFP was < 50 ng/mL, recurrent HCC occurred in 11% of patients (CI: 0.10–0.13). In comparison, studies with a mean AFP ≥ 50 ng/mL yielded a pooled prevalence of 15% (CI: 0.10–0.21).

### 3.4. Analyses by Viral and Nonviral Aetiology

HBV was present in 1947 patients, and the pooled prevalence of HCC recurrence was 18% (CI: 0.11–0.27). HCV was the underlying aetiology for liver disease in 12,331 patients, and the overall prevalence of recurrent HCC in HCV patients was 11% (CI: 0.08–0.15). Comparing viral aetiologies, a recurrence was significantly more common in HBV (*p* = 0.05).

For nonviral aetiologies, nonalcoholic steatohepatitis (NASH) was the underlying diagnosis for end-stage liver disease in 1791 patients, and the pooled prevalence of HCC recurrence for this group was 8% (CI: 0.01–0.20). ALD was also found in a total of 1868 patients, and the pooled analysis revealed the prevalence of HCC recurrence to be 10% (CI: 0.05–0.17).

### 3.5. Analyses by Ethnicity

**Figure 4:**
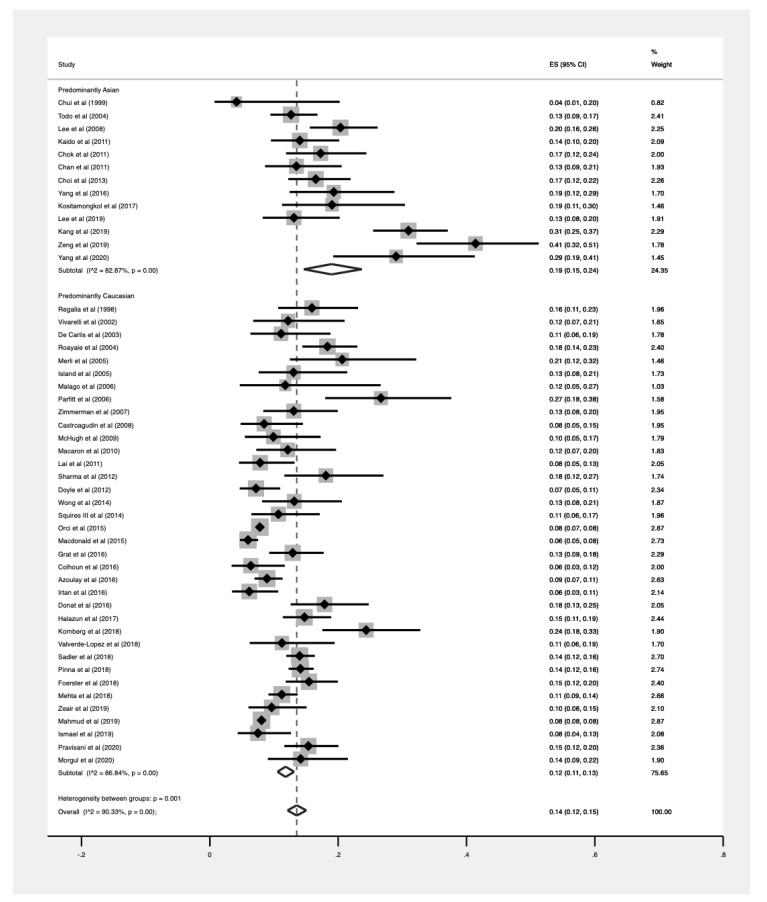
Pooled proportions stratified by Asian and Western populations.

The prevalences of HCC recurrence were pooled for Asian, Western, Middle Eastern, and Latin American populations and were found to be 19% (CI: 0.15–0.24), 12% (CI: 0.11–0.13), 16% (CI: 0.12–0.20), and 11% (CI: 0.09–0.14), respectively (Table 1). Comparing Asian and Western population subgroups, recurrent HCC had a significantly higher prevalence in Asian populations (*p* = 0.001, Figure 4).

Differences in the prevalence of recurrent HCC persisted even when some aetiologies were stratified by ethnicity. The two most commonly reported ethnicities were Asians and Caucasians. For patients with HBV, the analysis of 591 patients from the Asian HBV subgroup yielded a prevalence of 25% (0.15–0.37). The pooled analysis of 1343 patients from studies from Western countries revealed a prevalence of 11% (CI: 0.06–0.17). The prevalence of recurrent HCC in patients with underlying HBV was significantly increased in the Asian subgroup (*p* = 0.05).

Comparing prevalence in HCV patients stratified by ethnicity, Asian HCV patients had a prevalence of 12% (CI: 0.06–0.23), compared to Caucasian HCV patients with a prevalence of 12% (CI: 0.09–0.17). Interestingly, there was no statistically significant difference in prevalences between HCV patients of different ethnicities (*p* = 0.84).

### 3.6. Analyses by Milan Criteria

**Figure 5:**
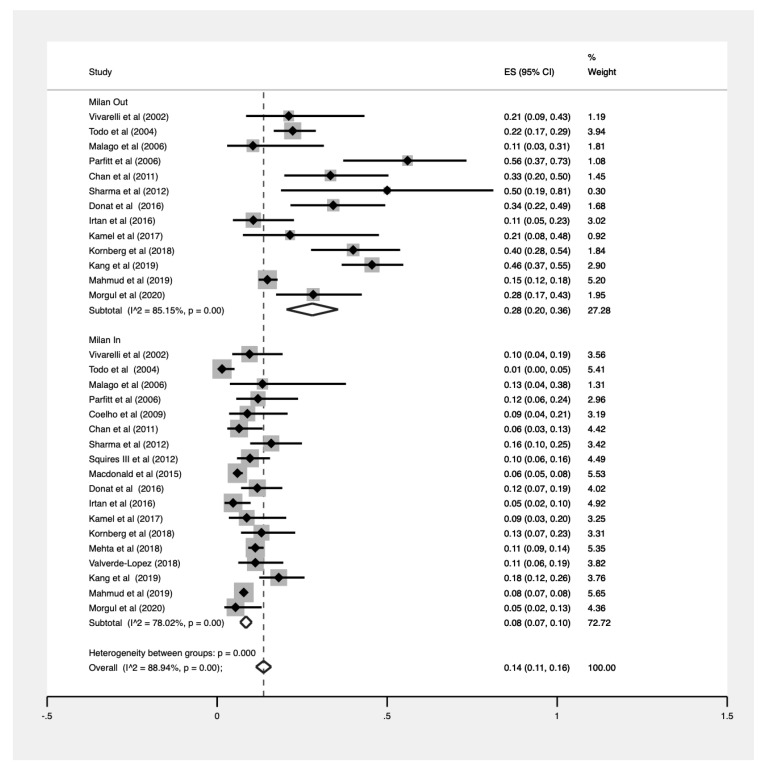
Pooled proportions stratified by the Milan criteria.

Additionally, 20,884 patients were within the Milan criteria (MC) prior to transplant, with the pooled prevalence of HCC recurrence for these patients being 8% (CI: 0.07–0.10, Figure 4). The analysis of patients beyond the MC yielded a pooled recurrence of 28% (CI: 0.20–0.36, Figure 4). There was a statistically significant difference in prevalences between patients within and beyond MC (*p* < 0.001, Figure 5).

### 3.7. Analysis by Deceased Donor Versus Living Donor Liver Transplant

**Figure 6:**
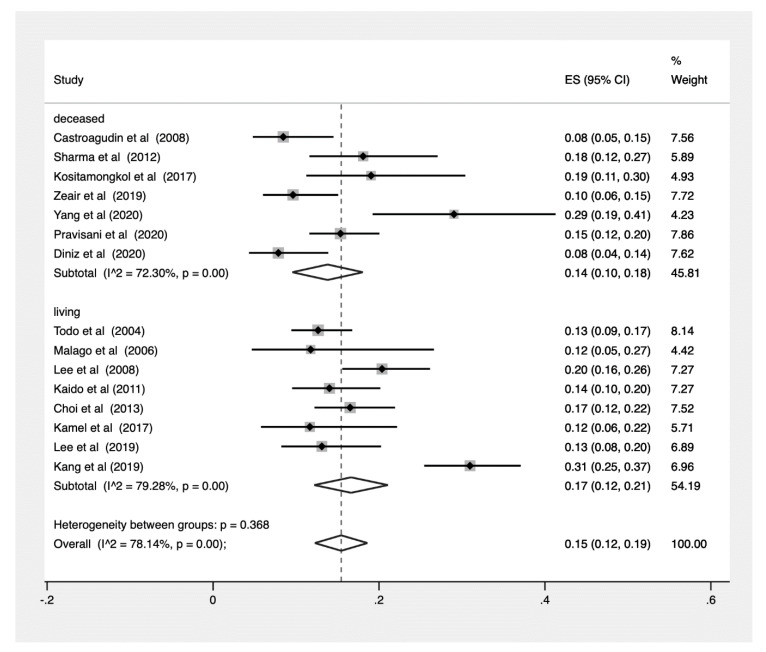
Pooled Proportions stratified by DDLT and LDLT.

The prevalence of hepatic recurrence was also pooled by living donor liver transplant (LDLT) compared to deceased donor liver transplant (DDLT) and was found to be 17% (CI: 0.12–0.21, Figure 5) and 14% (CI: 0.10–0.18, Figure 5), respectively. Comparing LDLT and DDLT, there was a nonsignificant difference in prevalences (*p* = 0.368, Figure 6).

### 3.8. Analysis by Income

The differences in HCC recurrences in patients from countries with varying economic levels were also evaluated. The pooled prevalences for middle-income and high-income subgroups were 15% (CI: 0.12–0.19) and 13% (CI: 0.11–0.14), respectively. There was a nonsignificant difference in the prevalences of recurrent HCC when comparing subgroups from different income levels (*p* = 0.17).

## 4. Discussion

When stringent selection criteria are adhered to [1], LT is known to have desirable long-term outcomes and is currently the gold standard for HCC treatment [9]. However, observational studies have found that post-transplant HCC recurrence is still common, estimated to be occurring in about 15–20% of cases [6,7,8]. Using a meta-analysis of proportions, the pooled prevalence of HCC recurrence was 13% (CI: 0.12–0.15) across 40,495 patients who underwent LT. However, the global trend of HCC recurrence seemed to be decreasing with time when a meta-regression was conducted on the year of study completion (β = −0.0015, SE: 0.002, *p* = 0.434, Figure 3). Recent improvements in the prevalence of HCC recurrence may be attributed to novel immunosuppressant strategies, including the use of mTOR inhibitors such as sirolimus and everolimus [28,29], and improved patient selection with stringent criteria [30]. Adherence to MC was a significant factor, with patients beyond MC experiencing HCC recurrence more frequently when compared to those within MC, which corroborated with previously existing meta-analyses [31]. The analysis was also pooled across different ethnicities, income levels, and underlying aetiologies for liver disease, with the prevalence of HCC recurrence found to be highest in Asian populations, among patients with HBV, and in middle-income countries.

In our analysis, the prevalence of recurrent HCC varied significantly based on ethnicity, with Asian populations having a significantly higher risk of recurrence compared to Western and Latin American populations. Although there is currently a paucity of literature reviewing the effects of ethnicity on HCC recurrence, these differences may be attributed in part to the varying management practices by region [32,33]. Notably, selection criteria differ across regions, with LDLT being the mainstay in several Asian countries due to a shortage of organs from deceased donors [34,35]. Patients undergoing LDLT may be subjected to less stringent selection criteria and thus possibly exceed MC and even other established criteria for transplantation [36], which correlates with the higher rate of HCC recurrence found in this study for those undergoing LDLT. Hence, the high prevalence of out-of-MC LDLTs within Asia could be a significant contributor to the higher recurrence in Asian populations. The increased prevalence of HCC recurrence in Asian populations may also be attributed to the underlying aetiology of liver disease, with HBV being the primary underlying cause of HCC in Asia [33,37,38] when compared to HCV infection in Western countries [39]. The disparity in the recurrence rate between different ethnicities even persisted when only HBV patients were analysed. Apart from the use of differing transplant criteria, genotypic variations in HBV may play a role, with Yuen et al. [40] reporting that HBV genotype C infection, which is endemic to East, South, and Southeast Asian populations [41], is associated with an increased viral load and consequently with more severe liver disease. The more frequent usage of LDLT in conjunction with the high disease burden of chronic HBV infection in Asia and genotypic differences may predispose the Asian population to a higher prevalence of HCC recurrence when compared to other ethnicities, as demonstrated in our analysis. However, data regarding the association of ethnicity with regional HCC recurrence must be interpreted with caution, as these classifications do not take into account the true racial and ethnic differences among groups.

The underlying aetiology of liver disease was also found to affect HCC recurrence. Our analysis revealed a higher prevalence of recurrence in patients with viral vs. nonviral aetiologies (18% and 11% for HBV and HCV respectively, compared to 10% and 8% for alcoholic liver disease and NASH respectively). The high prevalence of HCC recurrence in the HBV subgroup is a particular cause for concern. Recent evidence suggests benefits of anti-HBV prophylaxis and/or anti-HBV immunoglobulins in preventing post-transplant HBV recurrence [42,43], and these have been associated with an increased recurrence-free survival for HCC patients following LT [44]. However, further investigation is required due to a scarcity in reporting regarding the usage of anti-HBV therapy in LT patients in the studies included in this analysis.

Furthermore, NASH remains a clinically significant underlying aetiology despite a lower HCC recurrence in the NASH subgroup, with recent observational data reporting NASH as the fastest growing cause of HCC in LT patients within the United States [45]. Results from our analysis corroborate with a study by Lewin et al., which similarly reported a lower risk of HCC recurrence among patients with NASH when compared to non-NASH aetiologies [46,47]. However, the limited number of studies examining the differences between NASH and non-NASH aetiologies prompts the need for further research into this area.

Interestingly, the use of LRT before transplant, including transarterial chemoembolisation and radiofrequency ablation, did not significantly reduce the prevalence of recurrent HCC. The existing literature suggests that bridging LRT before transplant improves overall survival, with lower post-transplant recurrence; however, these benefits are exclusive to patients who have achieved a complete pathological response (cPR) with no remaining viable tumor upon explant pathology [11,46]. The studies included in our analysis consisted of patients that had undergone LRT regardless of cPR status, contributing to a lack of significant improvement in post-transplant recurrence and corroborating the importance of achieving cPR in reducing recurrence.

In addition, when the analysis was stratified according to the mean pretransplant AFP levels, recurrence was found to be higher in studies with a mean AFP ≥ 50 ng/mL. Current guidelines suggest that pre-LT AFP does provide a prognostic value for outcomes after transplantation [9], although there is currently a lack of consensus on the cut-off value that should be considered due to the wide range in the existing literature [47,48]. AFP has also been suggested as a predictor for the successful downstaging of tumours prior to transplant [9,11], although a threshold has yet to be established, thus requiring further investigation.

To further investigate the effect of income on the prevalence of post-LT HCC recurrence, an analysis was done to compare HCC recurrence in middle- and high-income countries, with stratifications of income levels according to World Bank definitions [16]. Previous studies pertaining to HCC have associated lower personal income levels with a decreased access to healthcare providers and a poorer recurrence-free survival due to the late detection of cancer [48,49], prompting the need for comparisons between countries with different income levels. From our analysis, patients from middle-income countries are more likely to develop a recurrence when compared to high-income countries. However, due to income inequalities within each country and the fact that the income of the country is not represented by the individual’s income, further investigation is required on the association between an individual’s income and healthcare accessibility in relation to HCC recurrence.

## 5. Limitations

There are several limitations to our study. While we attempted to extract and analyse the effect of MC, the lack of individual reporting of recurrence in individual MC analyses results in a smaller pool analysis of the effects of MC, thus preventing adjustments for ethnicity, aetiology, and living donor vs. dead donor transplants. Furthermore, the analysis on ethnicity classified patients into predominant ethnic groups based on their country of origin. However, the study population might have consisted of other ethnicities within the country, although they were likely to be in the minority. This analysis was also unable to account for other risk factors, including serum AFP levels, the use of locoregional therapies with response while waiting for transplant, the experience of transplantation centres, the vascular invasion status, tumour differentiation, and the adherence to other commonly used criteria beyond the Milan criteria due to a lack of extractable data and/or a scarcity of reporting. Furthermore, there was a significant heterogeneity in the reporting of immunosuppression due to varied immunosuppressive regimens within the same cohort and several patients discontinuing therapy, and we were unable to account for this in our analysis. As the results from current cohorts mature, future research regarding the influence of factors such as centre experience, locoregional therapy [11], and immunosuppression [32,33] on post-transplant HCC recurrence could be explored for a more comprehensive analysis. Finally, while NASH has been present in patient populations, it was often denoted as cryptogenic cirrhosis and was only classified as NASH during the transition period from 2000 to 2014 [50]. Hence, cryptogenic cirrhosis was classified as NASH in this article, although other idiopathic causes may also have been classified as cryptogenic in the included papers [51].

## 6. Conclusions

In conclusion, this meta-analysis revealed the overall prevalence of HCC recurrence to be 13%, with a higher recurrence observed in Asians, patients with HBV, and middle-income countries. Further studies are required to study the reasons behind the differences in recurrence among these subgroups. These findings provide useful guidance for clinicians counselling patients being considered for liver transplantation and will aid patient counselling for various populations with varying risk levels for post-transplant HCC recurrence. Further studies are required to discern the reason behind the disparities in HCC recurrence between ethnic groups and aetiologies.

## Figures and Tables

**Table 1:** Summary of the pooled prevalence of HCC recurrence.

	No. of Papers	Count	Total Sample Size	Pooled Prevalence (CI)
Overall Prevalence	58	3888	40,495	13% (0.12–0.15)
Alpha-Fetoprotein				
<50 ng/mL	17	3012	34,488	11% (0.10–0.13)
≥50 ng/mL	7	225	1491	15% (0.10–0.21)
Transplant Type ^a^				
Living donor	8	246	1380	17% (0.12–0.21)
Deceased donor	7	127	922	14% (0.10–0.18)
Milan Criteria ^a^				
Within Milan Criteria	18	1663	20,884	8% (0.07–0.10)
Beyond Milan Criteria	18	269	1199	28% (0.20–0.36)
Income				
Middle income	13	439	2908	15% (0.12–0.19)
High income	45	3449	37,587	13% (0.11–0.14)
Ethnicity				
Predominantly Asian	13	365	1887	19% (0.15–0.24)
Predominantly Western	36	3327	37,142	12% (0.11–0.13)
Predominantly Middle Eastern	3	66	412	16% (0.12–0.20)
Predominantly Latin American	6	130	1054	11% (0.09–0.14)
Aetiology ^a^				
HBV	13	277	1947	18% (0.11–0.27)
HCV	12	1037	12,331	11% (0.08–0.15)
NASH	7	125	1791	8% (0.01–0.20)
ALD	7	134	1868	10% (0.05–0.17)

Abbreviations: HCC = hepatocellular carcinoma; HBV = hepatitis B virus; HCV = hepatitis C virus; NASH = non-alcoholic steatohepatitis; ALD = alcoholic liver disease; Legend: ^a^ Not inclusive of total cohort size.

## Data Availability

All data available upon request.

## Supplementary Materials

The following are available online at https://www.mdpi.com/2077-0383/10/2/238/s1. Table S1. Medline search strategy; Table S2. Summary of key characteristics of included articles.
