# Supplementary material for: A Meta-Analysis on the Rate of Hepatocellular Carcinoma Recurrence after Liver Transplant and Associations to Etiology, Alpha-Fetoprotein, Income and Ethnicity
Source: J Clin Med. 2021 Jan 11;10(2):238. doi: 10.3390/jcm10020238 (PMC7828059; doi:10.3390/jcm10020238)
Supplement: Supplementary file 1 [file jcm-10-00238-s001.zip › jcm-1023146-SI/jcm-1023146-Table S1.docx]

**Table S1.** Medline search strategy.

| **No.** | **Search Input** |
| --- | --- |
| 1 | exp Liver Neoplasms/ |
| 2 | exp Carcinoma, Hepatocellular/ |
| 3 | ((hepat * OR liver *) adj3 (carcinoma * OR tumour * OR tumor * OR neoplasm * OR malign * OR cancer *)).tw. OR (HCC).tw. |
| 4 | 1 OR 2 OR 3 |
| 5 | exp Liver Transplantation/ |
| 6 | ((liver * OR hepat *) adj3 (transplan * OR graft *)).tw. |
| 7 | 5 OR 6 |
| 8 | exp recurrence/ |
| 9 | (recur * OR relaps *).tw. |
| 10 | 8 OR 9 |
| 11 | exp Incidence/ |
| 12 | exp prevalence/ |
| 13 | exp epidemiology/ |
| 14 | exp epidemiologic studies/ |
| 15 | (inciden * OR prevalen * OR epidemiol * OR occurrence).tw. |
| 16 | 11 OR 12 OR 13 OR 14 OR 15 |
| 17 | 4 AND 7 AND 10 AND 16 |
