# Supplementary material for: A Meta-Analysis on the Rate of Hepatocellular Carcinoma Recurrence after Liver Transplant and Associations to Etiology, Alpha-Fetoprotein, Income and Ethnicity
Source: J Clin Med. 2021 Jan 11;10(2):238. doi: 10.3390/jcm10020238 (PMC7828059; doi:10.3390/jcm10020238)
Supplement: Supplementary file 1 [file jcm-10-00238-s001.zip › jcm-1023146-SI/jcm-1023146-Table S2.docx]

**Table S2.** Summary of Key Characteristics of Included Articles.

| **Author** | **Year** | **Country** | **Mean Follow-up/Months** | **Patients with HCC Recurrence/*n*** | **Total Study Population/*n*** | **Diagnosis of HCC Recurrence** | **Quality Assessment—Risk of Bias** |
| --- | --- | --- | --- | --- | --- | --- | --- |
| Diniz et al [1] | 2020 | Brazil | 51.0 | 10 | 127 |  | Low |
| Morgul et al [2] | 2020 | Germany |  | 17 | 120 | Radiological findings + AFP | Low |
| Pravisani et al [3] | 2020 | Italy |  | 43 | 280 | Radiological findings | Low |
| Yang et al [4] | 2020 | China | 36.9 | 18 | 62 | Radiological findings | Low |
| Ismael et al [5] | 2019 | US |  | 12 | 160 | Radiological findings + AFP | Low |
| Kang et al [6] | 2019 | Korea | 53.0 | 74 | 239 | Radiological findings | Low |
| Lee et al [7] | 2019 | Korea | 71.3 | 16 | 122 | Radiological findings + AFP | Low |
| Mahmud et al [8] | 2019 | US |  | 1,484 | 18,406 | Radiological findings | Low |
| Polat et al [9] | 2019 | Turkey | 27.8 | 23 | 165 | Radiological findings + AFP | Low |
| Zeair et al [10] | 2019 | Poland |  | 16 | 166 | Radiological findings | Low |
| Zeng et al [11] | 2019 | China | 33.6 | 41 | 99 |  | Low |
| Foerster et al [12] | 2018 | Germany |  | 47 | 304 | Radiological findings | Low |
| Kornberg et al [13] | 2018 | Germany | 84.3 | 29 | 119 | Radiological findings | Low |
| Mehta et al [14] | 2018 | US | 46.8 | 83 | 740 |  | Low |
| Pinna et al [15] | 2018 | Italy, China |  | 172 | 1,218 |  | Moderate |
| Sadler et al [16] | 2018 | US | 55.9 | 130 | 929 | Radiological findings | Low |
| Valverde-Lopez et al [17] | 2018 | Spain |  | 10 | 89 |  | Low |
| Yilmaz et al [18] | 2018 | Turkey |  | 36 | 187 |  | Low |
| Halazun et al [19] | 2017 | US |  | 50 | 339 | Radiological findings | Low |
| Kamel et al [20] | 2017 | Egypt | 51.3 | 7 | 60 | Radiological findings + AFP | Low |
| Kositamongkol et al [21] | 2017 | Thailand |  | 12 | 63 |  | Low |
| Pinero et al [22] | 2017 | Latin America | 38.2 | 62 | 435 | Radiological findings + AFP | Low |
| Azoulay et al [23] | 2016 | France |  | 58 | 651 | Radiological findings + AFP | Low |
| Boteon et al [24] | 2016 | Brazil | 47.4 | 10 | 101 | Radiological findings | Low |
| Colhoun et al [25] | 2016 | US |  | 9 | 141 | Radiological findings | Moderate |
| Donat et al [26] | 2016 | Spain | 67.4 | 27 | 151 |  | Low |
| Grat et al [27] | 2016 | Poland | 34.0 | 31 | 240 |  | Low |
| Irtan et al [28] | 2016 | France | 65.0 | 11 | 179 | Radiological findings | Moderate |
| Schraiber et al [29] | 2016 | Brazil | 49.8 | 32 | 206 | Radiological findings | Low |
| Yang et al [30] | 2016 | Korea | 33.8 | 17 | 88 | Radiological findings | Low |
| Macdonald et al [31] | 2015 | US |  | 64 | 1,074 |  | Low |
| Orci et al [32] | 2015 | Switzerland |  | 758 | 9,724 |  | Low |
| Costa et al [33] | 2014 | Brazil |  | 12 | 140 | Radiological findings + AFP | Low |
| Squires III et al [34] | 2014 | US | 30.0 | 14 | 131 |  | Low |
| Wong et al [35] | 2014 | US |  | 15 | 114 | Radiological findings | Low |
| Choi et al [36] | 2013 | Korea | 70.2 | 37 | 224 | Radiological findings + AFP | Low |
| Doyle et al [37] | 2012 | US |  | 19 | 264 | Radiological findings + AFP | Low |
| Sharma et al [38] | 2012 | US |  | 17 | 94 | Radiological findings + AFP | Low |
| Chan et al [39] | 2011 | Taiwan | 35.2 | 17 | 126 | Radiological findings + AFP | Low |
| Chok et al [40] | 2011 | Hong Kong, China |  | 24 | 139 | Radiological findings + AFP | Low |
| Kaido et al [41] | 2011 | Japan |  | 23 | 164 |  | Low |
| Lai et al [42] | 2011 | Italy | 64.3 | 12 | 153 |  | Low |
| Macaron et al [43] | 2010 | US | 21.8 | 13 | 107 | Radiological findings | Low |
| Coelho et al [44] | 2009 | Brazil |  | 4 | 45 |  | Low |
| McHugh et al [45] | 2009 | US | 41.0 | 10 | 101 |  | Low |
| Castroagudin et al [46] | 2008 | Spain | 41.3 | 11 | 130 |  | Moderate |
| Lee et al [47] | 2008 | Korea | 43.0 | 45 | 221 | Radiological findings + AFP | Low |
| Zimmerman et al [48] | 2007 | US | 34.0 | 17 | 130 | Radiological findings | Low |
| Malago et al [49] | 2006 | Germany | 32.8 | 4 | 34 | Radiological findings + AFP | Low |
| Parfitt et al [50] | 2006 | Canada |  | 20 | 75 |  | Low |
| Island et al [51] | 2005 | US |  | 12 | 92 | Radiological findings | Low |
| Merli et al [52] | 2005 | Italy |  | 13 | 63 | Radiological findings + AFP | Low |
| Roayaie et al [53] | 2004 | US | 21.6 | 57 | 311 | Radiological findings + AFP | Low |
| Todo et al [54] | 2004 | Japan | 26.6 | 40 | 316 |  | Low |
| De Carlis et al [55] | 2003 | Italy |  | 11 | 99 | Radiological findings | Low |
| Vivarelli et al [56] | 2002 | Italy |  | 10 | 82 | Radiological findings + AFP | Low |
| Chui et al [57] | 1999 | Hong Kong, China | 32.7 | 1 | 24 |  | Low |
| Regalia et al [58] | 1998 | Italy | 10.4 | 21 | 132 | Radiological findings + AFP | Low |

References

1. Diniz PHC, Silva SDDC, Faria LC, Vidigal PVT, Ferrari TCA. Clinical and laboratory parameters as predictors of long-term outcome according to the etiology of underlying chronic liver disease in patients who underwent liver transplantation for hepatocellular carcinoma treatment. *Clinics (Sao Paulo, Brazil).* 2020;75:e1529.

2. Morgul MH, Felgendreff P, Kienlein A, et al. Milan criteria in the MELD era-is it justifiable to extend the limits for orthotopic liver transplantation? *World journal of surgical oncology.* 2020;18(1):158.

3. Pravisani R, Mocchegiani F, Isola M, et al. Controlling Nutritional Status score does not predict patients' overall survival or hepatocellular carcinoma recurrence after deceased donor liver transplantation. *Clinical Transplantation.* 2020;34(3).

4. Yang Z, Zhu H, Zhang L, et al. DNA methylation of SOCS1/2/3 predicts hepatocellular carcinoma recurrence after liver transplantation. *Molecular Biology Reports.* 2020;47(3):1773-1782.

5. Ismael MN, Forde J, Milla E, Khan W, Cabrera R. Utility of inflammatory markers in predicting hepatocellular carcinoma survival after liver transplantation. *BioMed Research International.* 2019;2019.

6. Kang YK, Choi JY, Paeng JC, et al. Composite criteria using clinical and FDG PET/CT factors for predicting recurrence of hepatocellular carcinoma after living donor liver transplantation. *European Radiology.* 2019;29(11):6009-6017.

7. Lee S, Kim KW, Jeong WK, et al. Gadoxetic acid–enhanced MRI as a predictor of recurrence of HCC after liver transplantation. *European Radiology.* 2020;30(2):987-995.

8. Mahmud N, Shaked A, Olthoff KM, Goldberg DS. Differences in Posttransplant Hepatocellular Carcinoma Recurrence by Etiology of Liver Disease. *Liver Transplantation.* 2019;25(3):388-398.

9. Polat KY, Acar S, Gencdal G, et al. Hepatocellular Carcinoma and Liver Transplantation: A Single-Center Experience. *Transplantation Proceedings.* 2020;52(1):259-264.

10. Zeair S, Rajchert J, Stasiuk R, et al. Recurrence of Hepatocellular Carcinoma After Liver Transplantation: A Single-Center Experience. *Annals of Transplantation.* 2019;24:499-505.

11. Zeng KN, Zhang YC, Wang GS, et al. A scoring model based on plasma fibrinogen concentration for predicting recurrence of hepatocellular carcinoma after liver transplantation. *Liver Research.* 2019;3(3-4):234-239.

12. Foerster F, Mittler J, Darstein F, et al. Recipient liver function before liver transplantation influences post-transplantation survival in patients with HCC. *European Journal of Internal Medicine.* 2018;55:57-65.

13. Kornberg A, Schernhammer M, Kornberg J, Friess H, Thrum K. Serological Risk Index Based on Alpha-Fetoprotein and C-Reactive Protein to Indicate Futile Liver Transplantation Among Patients with Advanced Hepatocellular Carcinoma. *Digestive Diseases & Sciences.* 2019;64(1):269-280.

14. Mehta N, Heimbach J, Lee D, et al. Wait Time of Less Than 6 and Greater Than 18 Months Predicts Hepatocellular Carcinoma Recurrence After Liver Transplantation: Proposing a Wait Time "Sweet Spot". *Transplantation.* 2017;101(9):2071-2078.

15. Pinna AD, Yang T, Mazzaferro V, et al. Liver transplantation and hepatic resection can achieve cure for hepatocellular carcinoma. *Annals of Surgery.* 2018;268(5):868-875.

16. Sadler EM, Mehta N, Bhat M, et al. Liver Transplantation for NASH-Related Hepatocellular Carcinoma Versus Non-NASH Etiologies of Hepatocellular Carcinoma. *Transplantation.* 2018;102(4):640-647.

17. Valverde-Lopez F, Angeles Lopez Garrido M, Ortega-Suazo EJ, Vadillo-Calles F, Muffak-Granero K, Nogueras-Lopez F. Results of 15-Year Experience in Liver Transplant for Hepatocellular Carcinoma. *Transplantation Proceedings.* 2018;50(2):617-618.

18. Yilmaz C, Karaca CA, Iakobadze Z, et al. Factors Affecting Recurrence and Survival After Liver Transplantation for Hepatocellular Carcinoma. *Transplantation Proceedings.* 2018;50(10):3571-3576.

19. Halazun KJ, Najjar M, Abdelmessih RM, et al. Recurrence After Liver Transplantation for Hepatocellular Carcinoma: A New MORAL to the Story. *Annals of Surgery.* 2017;265(3):557-564.

20. Kamel R, Hatata Y, Hosny K, et al. Outcome of Living-Donor Liver Transplant for Hepatocellular Carcinoma: 15-Year Single-Center Experience in Egypt. *Experimental & Clinical Transplantation: Official Journal of the Middle East Society for Organ Transplantation.* 2017;15(Suppl 2):12-20.

21. Kositamongkol P, Sanphasitvong V, Sirivatanauksorn Y, et al. Outcome of Liver Transplantation in Hepatocellular Carcinoma Patients at Siriraj Hospital. *Transplantation Proceedings.* 2017;49(5):1114-1117.

22. Piñero F, Tisi Baña M, de Ataide EC, et al. Liver transplantation for hepatocellular carcinoma: evaluation of the alpha-fetoprotein model in a multicenter cohort from Latin America. *Liver International.* 2016;36(11):1657-1667.

23. Azoulay D, Audureau E, Bhangui P, et al. Living or Brain-dead Donor Liver Transplantation for Hepatocellular Carcinoma. *Annals of Surgery.* 2017;266(6):1035-1044.

24. Boteon YL, Carvalheiro da Silva AP, Boin IF, de Ataide EC. Evaluation of Recurrence Predictors and Survival Probability After Liver Transplantation for Hepatocellular Carcinoma: Analysis From a Single Center. *Transplantation Proceedings.* 2016;48(6):2087-2093.

25. Colhoun EDt, Forsberg CG, Chavin KD, Baliga PK, Taber DJ. Incidence and risk factors of hepatocellular carcinoma after orthotopic liver transplantation. *Surgery.* 2017;161(3):830-836.

26. Donat M, Alonso S, Pereira F, et al. Impact of Histological Factors of Hepatocellular Carcinoma on the Outcome of Liver Transplantation. *Transplantation Proceedings.* 2016;48(6):1968-1977.

27. Grat M, Wronka KM, Stypulkowski J, et al. The Warsaw Proposal for the Use of Extended Selection Criteria in Liver Transplantation for Hepatocellular Cancer. *Annals of Surgical Oncology.* 2017;24(2):526-534.

28. Irtan S, Barbier L, Francoz C, Dondero F, Durand F, Belghiti J. Liver transplantation for hepatocellular carcinoma: is zero recurrence theoretically possible? *Hepatobiliary & Pancreatic Diseases International.* 2016;15(2):147-151.

29. Schraiber Ldos S, de Mattos AA, Zanotelli ML, et al. Alpha-fetoprotein Level Predicts Recurrence After Transplantation in Hepatocellular Carcinoma. *Medicine.* 2016;95(3):e2478.

30. Yang K, Lee TB, Choi BH, et al. Development and Applicability of the A-P 200 Criteria for Liver Transplantation for Hepatocellular Carcinoma. *Transplantation Proceedings.* 2016;48(10):3317-3322.

31. Macdonald B, Sewell JL, Harper AM, Roberts JP, Yao FY. Liver transplantation for hepatocellular carcinoma: analysis of factors predicting outcome in 1074 patients in OPTN Region 5. *Clinical Transplantation.* 2015;29(6):506-512.

32. Orci LA, Berney T, Majno PE, et al. Donor characteristics and risk of hepatocellular carcinoma recurrence after liver transplantation. *British Journal of Surgery.* 2015;102(10):1250-1257.

33. Costa PEG, Vasconcelos JBM, Coelho GR, et al. Ten-year experience with liver transplantation for hepatocellular carcinoma in a Federal University Hospital in the Northeast of Brazil. *Transplantation Proceedings.* 2014;46(6):1794-1798.

34. Squires MH, 3rd, Hanish SI, Fisher SB, et al. Transplant versus resection for the management of hepatocellular carcinoma meeting Milan Criteria in the MELD exception era at a single institution in a UNOS region with short wait times. *Journal of Surgical Oncology.* 2014;109(6):533-541.

35. Wong RJ, Wantuck J, Valenzuela A, et al. Primary surgical resection versus liver transplantation for transplant-eligible hepatocellular carcinoma patients. *Digestive Diseases & Sciences.* 2014;59(1):183-191.

36. Choi HJ, Kim DG, Na GH, Han JH, Hong TH, You YK. Clinical outcome in patients with hepatocellular carcinoma after living-donor liver transplantation. *World Journal of Gastroenterology.* 2013;19(29):4737-4744.

37. Doyle MBM, Vachharajani N, Maynard E, et al. Liver transplantation for hepatocellular carcinoma: Long-term results suggest excellent outcomes. *Journal of the American College of Surgeons.* 2012;215(1):19-28.

38. Sharma P, Welch K, Hussain H, et al. Incidence and risk factors of hepatocellular carcinoma recurrence after liver transplantation in the MELD era. *Digestive Diseases & Sciences.* 2012;57(3):806-812.

39. Chan KM, Chou HS, Wu TJ, Lee CF, Yu MC, Lee WC. Characterization of hepatocellular carcinoma recurrence after liver transplantation: perioperative prognostic factors, patterns, and outcome. *Asian Journal of Surgery.* 2011;34(3):128-134.

40. Chok KS, Chan SC, Cheung TT, Chan AC, Fan ST, Lo CM. Late recurrence of hepatocellular carcinoma after liver transplantation. *World Journal of Surgery.* 2011;35(9):2058-2062.

41. Kaido T, Mori A, Ogura Y, et al. Recurrence of hepatocellular carcinoma after living donor liver transplantation: what is the current optimal approach to prevent recurrence? *World Journal of Surgery.* 2011;35(6):1355-1359.

42. Lai Q, Avolio AW, Manzia TM, et al. Role of alpha-fetoprotein in selection of patients with hepatocellular carcinoma waiting for liver transplantation: must we reconsider it? *International Journal of Biological Markers.* 2011;26(3):153-159.

43. Macaron C, Hanouneh IA, Lopez R, Aucejo F, Zein NN. Total tumor volume predicts recurrence of hepatocellular carcinoma after liver transplantation in patients beyond Milan or UCSF criteria. *Transplantation Proceedings.* 2010;42(10):4585-4592.

44. Coelho GR, Vasconcelos KF, Vasconcelos JB, et al. Orthotopic liver transplantation for hepatocellular carcinoma: one center's experience in the Northeast of Brazil. *Transplantation Proceedings.* 2009;41(5):1740-1742.

45. McHugh PP, Gilbert J, Vera S, Koch A, Ranjan D, Gedaly R. Alpha-fetoprotein and tumour size are associated with microvascular invasion in explanted livers of patients undergoing transplantation with hepatocellular carcinoma. *HPB.* 2010;12(1):56-61.

46. Castroagudin JF, Molina E, Bustamante M, et al. Orthotopic liver transplantation for hepatocellular carcinoma: a thirteen-year single-center experience. *Transplantation Proceedings.* 2008;40(9):2975-2977.

47. Lee SG, Hwang S, Moon DB, et al. Expanded indication criteria of living donor liver transplantation for hepatocellular carcinoma at one large-volume center. *Liver Transplantation.* 2008;14(7):935-945.

48. Zimmerman MA, Trotter JF, Wachs M, et al. Predictors of long-term outcome following liver transplantation for hepatocellular carcinoma: a single-center experience. *Transplant International.* 2007;20(9):747-753.

49. Malagó M, Sotiropoulos GC, Nadalin S, et al. Living donor liver transplantation for hepatocellular carcinoma: A single-center preliminary report. *Liver Transplantation.* 2006;12(6):934-940.

50. Parfitt JR, Marotta P, Alghamdi M, et al. Recurrent hepatocellular carcinoma after transplantation: use of a pathological score on explanted livers to predict recurrence. *Liver Transplantation.* 2007;13(4):543-551.

51. Island ER, Pomposelli J, Pomfret EA, Gordon FD, Lewis WD, Jenkins RL. Twenty-year experience with liver transplantation for hepatocellular carcinoma. *Archives of Surgery.* 2005;140(4):353-358.

52. Merli M, Nicolini G, Gentili F, et al. Predictive factors of outcome after liver transplantation in patients with cirrhosis and hepatocellular carcinoma. *Transplantation Proceedings.* 2005;37(6):2535-2540.

53. Roayaie S, Schwartz JD, Sung MW, et al. Recurrence of hepatocellular carcinoma after liver transplant: patterns and prognosis. *Liver Transplantation.* 2004;10(4):534-540.

54. Todo S, Furukawa H, Matsushita M, et al. Liver transplantation for patients with hepatitis B/C virus cirrhosis or hepato cellular carcinoma. *Nippon Geka Gakkai zasshi.* 2002;103(5):408-413.

55. De Carlis L, Giacomoni A, Lauterio A, et al. Liver transplantation for hepatocellular cancer: Should the current indication criteria be changed? *Transplant International.* 2003;16(2):115-122.

56. Vivarelli M, Bellusci R, Cucchetti A, et al. Low recurrence rate of hepatocellular carcinoma after liver transplantation: better patient selection or lower immunosuppression? *Transplantation.* 2002;74(12):1746-1751.

57. Chui AK, Rao AR, McCaughan GW, et al. Liver transplantation for hepatocellular carcinoma in cirrhotic patients. *Australian & New Zealand Journal of Surgery.* 1999;69(11):798-801.

58. Regalia E, Fassati LR, Valente U, et al. Pattern and management of recurrent hepatocellular carcinoma after liver transplantation. *Journal of Hepato-Biliary-Pancreatic Surgery.* 1998;5(1):29-34.
